# A single‐sided trans‐nasal catheter for smooth and efficient oxygen delivery can improve the safety in patients undergoing pancreato‐biliary endoscopy under intravenous sedation: A randomized trial

**DOI:** 10.1002/deo2.130

**Published:** 2022-06-12

**Authors:** Ken Ishii, Kensuke Kubota, Tomomi Nakao, Yuji Koyama, Yuji Fujita, Kuniaki Akaba, Nobuyuki Matsuhashi, Atsushi Nakajima

**Affiliations:** ^1^ Department of Gastroenterology National Hospital Organization Yokohama Medical Center Kanagawa Japan; ^2^ Department of Gastroenterology and Hepatology Yokohama City University, Graduate School of Medicine Kanagawa Japan; ^3^ Department of Gastroenterology Kanto Rosai Hospital Kanagawa Japan; ^4^ Department of Hepato Biliary Pancreatic Medicine NTT Medical Center Tokyo Tokyo Japan; ^5^ St. Marianna University School of Medicine Otolaryngology Kanagawa Japan

**Keywords:** dead space in the upper airway, intravenous sedation, pancreato‐biliary endoscopy, randomized trial, single‐sided trans‐nasal catheter

## Abstract

**Background:**

Pancreato‐biliary endoscopic procedures often need to be performed under deep intravenous sedation. The patients are at an increased risk of respiratory depression influenced by the anatomical dead space of the upper respiratory system. We aimed to evaluate the benefit of oxygen delivery through a single‐sided trans‐nasal catheter (TC) for patients undergoing pancreato‐biliary endoscopy.

**Methods:**

Oxygen supplementation during the procedure was provided either by insertion of a single‐sided TC or insertion of a conventional nasal catheter (NC). A prospective, single‐blind, randomized controlled study was conducted in two groups.

**Results:**

The number of patients who indicated a decrease in the peripheral transcutaneous oxygen saturation (SpO2; desaturation) was significantly lower in the TC group than in the counterpart (8/58; 13.8% vs. 26/58; 44.8% *p* < 0.001). The efficient oxygen delivery in the safe range was better conserved in the TC group than in the NC one. There was no adverse effect on both groups. The maximum SpO2 while the endoscopic procedure was significantly higher in the TC group (99.7% vs. 99.3% *p* = 0.016) and the minimum SpO2 was also significantly higher in the same group (97.7% vs. 94.1% *p* < 0.0001), which meant that the efficient oxygen delivery was better maintained in TC group than the NC group.

**Conclusions:**

A single‐sided TC placed in the pharynx in patients undergoing pancreato‐biliary endoscopy prepares a superior condition of the patients for venous sedation, maintained hyper‐oxygen saturation and a relatively higher SpO2 level to be maintained in limited conditions to reduce the dead space with acceptable tolerance, as compared to the placement of a conventional NC.

## INTRODUCTION

The type/depth of sedation used during endoscopic procedures depends on the discretion/skill level of the endoscopists; mainly intravenous sedation is used in spontaneously breathing patients, while general anesthesia is used in a small percentage of patients.^1^ Technically demanding pancreato‐biliary endoscopy (PBE), compared with another upper gastrointestinal endoscopy, often require deep sedation with an increment dose of sedatives for the patients, which entails an increased risk of respiratory depression, airway obstruction, hemodynamic instability, and/or respiratory arrest.^2^ Under the circumstance, the standard method of oxygen delivery in Japan, namely, nasal oxygen delivery via a standard nasal catheter (NC), may be of limited value in patients with upper airway obstruction including nasal, oral cavity, and pharynx cavities,^3^ and may be inadequate to correct hypoxemia in patients undergoing PBE with the above complications, because of the presence of anatomical dead space (DS) in the nasal, oral and pharyngeal cavities that could undermine sufficient oxygen delivery.^4^ Furthermore, inadvertent oversedation could cause further respiratory depression and oxygen desaturation.^5^ On the other hand, supportive use of pharyngeal cannulas that are used in the field of pediatric anesthesia may be a suitable alternative to reduce DS caused not only by narrowing nasal cavity, the swollen pharynx, and larynx in some situations but it has not yet been used for adult patients in Japan. The temporality of oxygen desaturation under intravenous sedation could be prevented by nasal oxygen delivery at a flow rate of 2–4 L/min^6^ to maintain oxygen level above 90%. This method might deliver oxygen effectively if the catheter could be placed within the nasal cavity, commonly used for pediatric anesthesia.^7^ However, this single‐sided trans‐nasal catheter (TC) is not originally intended for oxygen administration and is not approved by the government without evidence. We hypothesized that this single‐sided trans‐nasal oxygen delivery catheter could decrease the incidence of hypoxia in adult patients who have mainly suffered from hypoxemia due to DS in the upper airway undergoing PBE with intravenous sedation.

## MATERIALS AND METHODS

This was a prospective, single‐blind, randomized controlled study conducted at NTT medical center Tokyo between April 2017 and July 2017 to evaluate the patient's safety and tolerance of a single‐sided TC compared with commonly used NC. This study protocol was approved by the NTT medical center Tokyo Institutional Review Board (ID: 77567), and the study was registered in the University Hospital Medical Information Network (UMIN ID: 000029269), Japan. Written informed consent was obtained from each of the participating patients. The patients who underwent PBE, such as endoscopic retrograde cholangiopancreatography (ERCP) and endoscopic ultrasonography (EUS), were randomized into the single‐sided TC and NC groups using the block method before the procedure. They were randomly assigned in a 1:1 ratio. The block size was four, which was confidential to the endoscopists. This random number list was developed by a statistic expert without involvement in this study. The allocated procedures were sealed in sequentially numbered obscure envelopes and assigned to each patient at the start of sedation. Participants and endoscopists who judge results were blinded to randomization. Eligible patients were both male and female ages over 20 years. Patients with severe respiratory failure requiring tracheal intubation, pregnancy, and pre‐procedure hemodynamic instability were excluded. This trial was performed in compliance with the CONSORT guidelines (www.consort‐statement.org.) for conducting randomized clinical trials.

### Intravenous sedative

Prior to the PBE, all participants have recorded an initial oxygen level at room air and administered midazolam (total dose; 2–15 mg, mean dose; 5.5 mg, 1–2 mg initially, and additional 1 mg doses) as the sedative concomitantly with pentazocine (total dose; 15–30 mg, mean dose; 15 mg, 7.5 mg initially with additional doses as required to maintain patient's safety) for pain relief, as endoscopist‐directed intravenous sedation by an independent endoscopist after providing oxygen at 2 L/min. The endoscopist also supervised the administration of the sedative in consultation with the operator. After evaluating the consciousness level to determine the sedation status based on the Ramsay score,^8^ the appropriate endoscopic procedure was carried out. If a patient awoke from anesthesia during the procedure, additional injections were administered, as appropriate. To allow the patients to be monitored, the patients were moved into the endoscopic unit and connected to standard monitors, including continuous electrocardiographic, automated blood pressure, and pulse oximetry monitoring. The baseline peripheral arterial oxygen saturation was measured using a pulse oximeter with a finger probe. If the oxygen saturation dropped below 90%, the endoscopists could intervene with measures such as increasing the rate of oxygen supplementation (the oxygen dose was incremated by 1 L/min until the oxygen saturation level could recover more than 90%), chin lift, jaw thrust, placement of a nasal or oral airway, or bag‐mask ventilation, to counter the drop in oxygen saturation.

### Selection of a proper TC

There were two sizes of NC (10 and 14 Fr) available in NTT medical center Tokyo at that time. Ten other voluntary members studied the feasibility of both with a paper written informed consent. The results showed that three out of 10 members suffered from nasal bleeding and the difficult insertion when it came to trying it using a 14 Fr catheter, while the use of 10 Fr one did not show any negative effect on them with maintaining sufficient oxygen delivery. Based on this experiment, the 10 Fr TC was chosen as a catheter in this RCT.

### Method of oxygen supplementation

**FIGURE 1:**
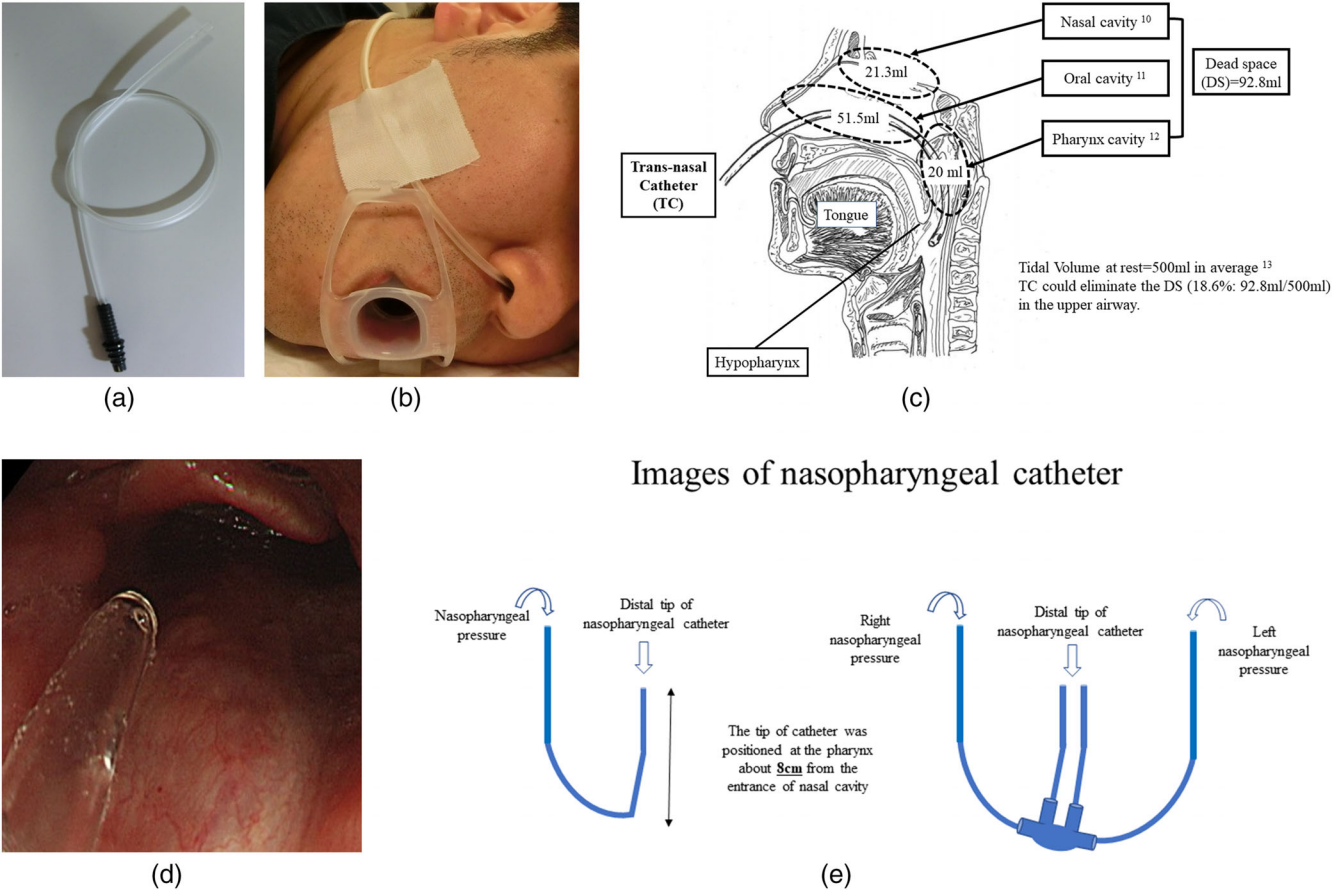
(a) A trans‐nasal catheter (Terumo Safeed nasal catheter 10 Fr; Terumo Japan. Co. with permission) (b). A single‐sided trans‐nasal catheter was placed. (c) A sagittal image of the pharyngeal section with trans‐nasal catheter placement and dead space in the upper airway. The dead space (DS) in the upper airway consists of three parts, such as nasal cavity (21.3 ml),^11^ oral cavity (51.5 ml),^12^ and pharynx cavity (20 ml).^13^ As the average tidal volume of an adult is 500 ml,^14^ a trans‐nasal catheter could reduce the DS. (18.6%: 92.8 ml/500 ml). (d) The end of the single‐sided trans‐nasal catheter was endoscopically visualized. (e) Images of a nasopharyngeal catheter. Left image indicated a nasopharyngeal catheter. It is commonly used for pediatric anesthesia. Right image showed a two‐sided trans‐nasal catheter

Patients in both groups received oxygen at 2 L/min through the cannula before they were sedated. 10 Fr single‐sided TC (Figure 1a) was adapted. The catheter was introduced into the hypopharynx so that the unfavorable influence of the upper respiratory DS, such as in the nasal, oral and pharyngeal cavities could be overcome (Figure 1b). The catheter was inserted about 8 cm into the pharynx from the nostril (Figure 1c). This allowed ideal oxygen delivery for the patient while allowing the drawback of the upper respiratory DS to be overcome. As the catheter position can be visualized and corrected during the endoscopy procedure (Figure 1d), the proper position of the catheter was attested before and after the procedure. Images of the nasopharyngeal catheter (NPC) were shown (Figure 1e).

**FIGURE 2:**
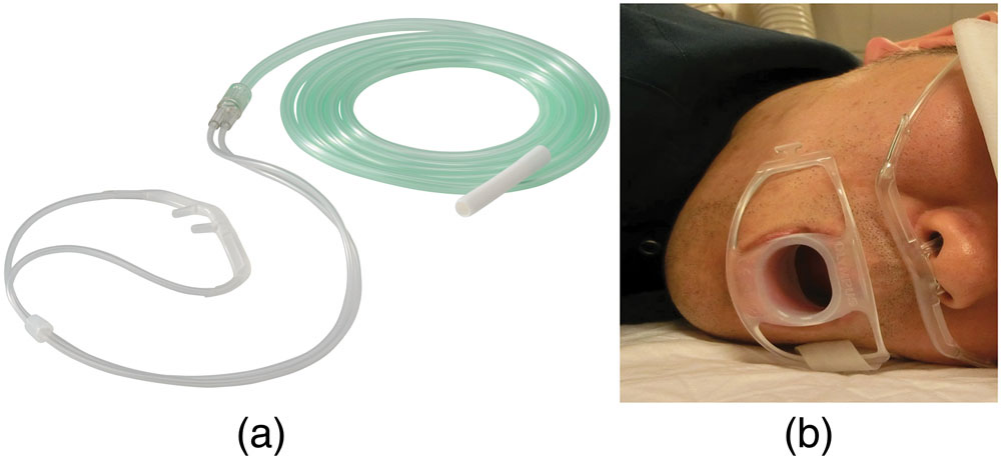
(a) A commonly used nasal catheter (Nasal oxygen catheter; Terumo Japan Co. with permission). (b) A nasal catheter was placed

The NC (Figure 2a) is fixed at the orifice of the nose to facilitate incremental oxygen supplementation of the inspired air (Figure 2b); however, overcoming the disadvantages of the upper respiratory DS is a challenge.

### The definition and judgment of SpO2 decrease

Procedure time is defined as the time between insertion of endoscopy and withdrawal of it. Desaturation (hypoxia) is defined as the drop of peripheral transcutaneous oxygen saturation (SpO2) over 5% for over 5 s.^9^ In the endoscopic room, there were two endoscopists (an operator and a monitor endoscopist) and one nurse, one of the endoscopists (a monitor endoscopist who was blinded to randomization), was present during the procedure to supervise the depth of sedation and to address the potential deterioration of the respiratory system. To assess the desaturation, SpO2 was studied through a SpO2 pulse oximetry monitor. Following induction, monitoring of the patient, control of sedation, and documentation including oxygen decrease <90% on pulse oximetry was conducted by this monitor endoscopist. More than a single period of oxygen desaturation was defined as SpO2 desaturation, which was recorded and assessed by this endoscopist. Standard monitoring such as heart rate, blood pressure, and SpO2 was continuously monitored and recorded in all patients. All adverse events that occurred in the procedure were also put down. As for the setting in case of desaturation, the increased flow was adjusted up to a maximum setting of 5 L/min.

### Distress to the awake patients

Consideration was made to the patients with Ramsay scores at 2 or 3.^8^ Under the situation, an additional sedative was given and confirmed whether the patient could tolerate to the endoscopic procedure by assuring their will and stable vital signs.

### Endpoints

The primary endpoint was the number of patients with desaturation in each group indicated by the SpO2 dropping more than 5% for more than 5 s during the procedure.

The secondary endpoint was the incidence due to side effects of placing a single‐sided TC or NC in the two groups within 24 h after the procedures (nasal bleeding, difficult insertion, postoperative discomfort in the pharynx or nose). Endpoints were judged and recorded by the monitor endoscopist.

## STATISTICAL ANALYSIS

Data are expressed as the means standard deviation or medians for continuous data, and as frequencies and percentages for categorical data. For continuous data, the characteristics of and outcomes in the two groups were compared by Student's t‐test or the Wilcoxon‐Mann‐Whitney U test, based on the viability of the normality assumption. Chi‐square or Fisher's exact test was used to compare two groups regarding the categoric variables. The level of significance was set at a two‐sided *p*‐value of 0.05. The DS in the upper airway consists of three parts, such as nasal cavity (21.3 ml),^10^ oral cavity (51.5 ml),^11^ and pharynx cavity (20 ml).^12^ As the average tidal volume of an adult is 500 ml,^13^ TC could reduce the DS (18.6%: 92.8 ml/500 ml) (Figure 1c). Therefore, this study was powered to detect a 19% difference in the number of desaturation events between the two study groups with 80% power (α = 5%). As a result, the target enrollment was 102 patients each for group TC (*n* = 51) and NC (*n* = 51). Since there was a concern that inserting a feeding catheter in the TC group might be discomfort for patients in this study, it is also typical of most trials, the required sample size was adjusted for an estimated dropout rate of 15% in this study. This extent of potential dropout would have been estimated from a previous study.^14^ As a result, there were 58 patients in each group. Statistical analysis was performed using BellCurve for Excel 2018 (Social Survey Research Information Co., Ltd., Japan).

## RESULTS

A total of 116 subjects were enrolled in this randomized controlled trial. There was no participant dropout in the study.

Table 1 shows the baseline characteristics of the study subjects. No significant differences were identified between the two patient groups: TC and NC groups, in the demographic or clinical characteristics, including the age, gender distribution, body mass index (BMI), the proportion of smokers, the proportion of patients with a history of excess alcohol consumption, percentage of patients with comorbidities such as respiratory diseases, neurological diseases, and so forth, and the American Society of Anesthesiologists score.^9^ Regarding the indication of endoscopy, most of the patients (around 90%) were conducted therapeutic ERCP and/or EUS‐guided drainage. There were no significant differences in the indication of PB endoscopy between the two groups. However, there was a higher proportion of patients with malignancy in the NC group as compared to that in the TC group.

**TABLE 1:** The baseline characteristics of study subjects

	TC (*n* = 58)	NC (*n* = 58)	*p*‐value
Age (years), mean ± SD [range]	71 ± 13 [49–89]	72 ± 13.6 [40–94]	0.717
Gender; male (%)	36 (62.1)	43 (74.1)	0.232
BMI (kg/m^2^), mean ± SD [range]	21.5 ± 3.9 [14.8–29.1]	22.2 ± 3.3 [11.8–34.3]	0.251
BMI (kg/m^2^), 30 or more (%)	0	1 (1.7)	1
Tobacco use (%)	4 (6.9)	8 (13.8)	0.238
Alcohol use (%)	17 (29.3)	20 (34.5)	0.691
Comorbidities			
Respiratory disorders (%)	4 (6.9)	7 (12.1)	0.238
Neurological disorders (%)	4 (6.9)	2 (3.5)	0.679
Indication of endoscopy			
Diagnostic ERCP	6 (10.3)	3 (4.9)	0.717
Therapeutic ERCP	50 (86.2)	47 (81.3)	0.442
EUS‐guided drainage	2 (3.5)	8 (13.8)	0.094
Malignant (%)	11 (18.9)	17 (29.3)	0.145
ASA classification			
1 (%)	25 (43.1)	17 (29.3)	0.21
2 (%)	23 (39.7)	31 (53.4)	0.22
3 (%)	9 (15.5)	10 (17.2)	0.64

Table 2 shows that there was no difference between the two groups in the mean starting SpO2 at the room air, the total midazolam dose used, the dose of pentazocine administered, or procedure time. However, the Ramsay score^8^ tended to be higher in the NC group as compared to that in the TC group. The number of patients who indicated a decrease in the SpO2 (desaturation) was significantly lower in the TC group than in the counterpart (8/58; 13.8% vs. 26/58; 44.8% *p* < 0.001). Most events of desaturation were transient less than 10 s and easily countered by airway intervention in the form of jaw thrust with an increment of oxygen by 1–2 L/min.

**TABLE 2:** Outcomes of patients who underwent pancreato‐biliary endoscopy in this trial

	TC (*n* = 58)	NC (*n* = 58)	*p*‐value
Procedure time, mean + SD [median:range]	33.7 ± 26.7 [3–130]	31.2 ± 20.4 [4–90]	*0.57*
Mean starting SpO2 (%), mean ± SD	99.8 ± 0.38	99.7 ± 0.44	0.263
Ramsay score			
2 (%)	1 (1.7)	1 (1.7)	1
3 (%)	1 (1.7)	2 (3.4)	0.56
4 (%)	38 (65.5)	31 (53.4)	0.26
5 (%)	18 (31)	26 (44.8)	0.23
Midazolam dose (mg), mean ± SD	6.0 ± 2.6	5.4 ± 2.2	0.17
Pentazocine dose (mg), mean ± SD	16.3 ± 4.2	15.5 ± 2.8	0.24
Procedure time (minutes), mean ± SD	33.7 ± 26.7	31.2 ± 20.4	0.57
Oxygen supplementation at start SpO2, %, mean ± SD	99.8 ± 0.2	99.5 ± 0.2	0.26
SpO2 decreased (%)	3 (5.2)	13 (22.4)	0.013
SpO2 dropped 5%< (%)	8 (13.8)	26 (44.8)	<0.001
SpO2 Maximum, %	99.7 ± 0.7	99.3 ± 1.2	0.016
SpO2 Minimum, %	97.7 ± 3.1	94.1 ± 6.2	<0.001
Nasal bleeding	0	0	1
Difficult insertion placement of catheter	0	0	1
Postoperative discomfort (pharynx, nose)	0	0	1

The maximum SpO2 while the endoscopic procedure was significantly higher in the TC group (99.7% vs. 99.3%, *p* = 0.016) and the minimum SpO2 was also significantly higher in the same group (97.7% vs. 94.1%, *p* < 0.0001), which meant that the efficient oxygen delivery was better maintained in TC group than the NC group.

Regarding secondary outcomes, there was no adverse effect on both groups. No dislocation of the stent was noted. There was one obesity, indicated BMI over 30 kg/m^2^, without desaturation in the NC group.

## DISCUSSION

This is the first study conducted to assess the usefulness of a single‐sided TC in patients undergoing mainly therapeutic PBE. As an introduction of this catheter into the pharyngeal prepares the patient for safe intravenous sedation and reduces hypoxemic events without any adverse effect, it allows higher SpO2 levels to be maintained by reducing DS in the upper airway than that compared to a conventional NC.

Nasal catheters have been commonly used for sedated endoscopies performed under intravenous anesthesia in Japan.^3^ However, there are drawbacks to using these catheters, including unfavorable anatomic factors, the patient's position, and the presence of the tongue and DS, which potentially interfere with smooth and sufficient oxygen delivery. Consequently, the SpO2 often plummeted during the endoscopic procedures. As a result, endoscopists in Japan still need to address the unexpected decrease in SpO2 level, while they must conduct endoscopic procedures with a high success rate. From these viewpoints, we consider the use of the single‐sided TC simple, safe, and cost‐effective.

Upfront use of the catheter could provide patients with a stable oxygen supply without the need for double catheterization, in which two TCs were inserted in both nasal cavities. As indicated in Table 2, the number of drops in the SpO2 decreased, and both the maximum and minimum SpO2 were maintained in the safe range; consequently, the fatality rate associated with the procedures performed under intravenous sedation/anesthesia could be minimized. Furthermore, the use of this single‐sided TC could be cost‐effective as compared to general anesthesia or the use of an NPC.^15^ Indeed, NPCs need to place as double catheters in the nose, which would be uncomfortable and hazardous for the patients during the procedure.

As for promising indicators of single‐sided trans‐nasal catheterization, patients who are obese or have sleep apnea, which can increase respiratory distress, could receive the benefit from this catheter. This method can maintain effective airway bypassed by the single‐sided TC when it comes to the patients with tongue root subsidence, while patients afflicted with increasing physiological DS such as chronic obstructive pulmonary disease would be unsuitable candidates for this catheter. Although Table 1 showed that there was only one obesity potentiated to sleep apnea^16^ in this study, this patient was not suffered desaturation. In these patients, alternatives, such as general anesthesia, which would be economically challenging, or the use of an NPC or high‐flow NC (HFNC) is preferable. Oxygen supplementation via an NPC in patients under intravenous general anesthesia was associated with significantly fewer episodes of hypoxemia and several airways assist maneuvers needed than the use of the traditional NC, however, NPCs are not available for use in adults receiving intravenous sedation in Japan. HFNCs could allow a constant FiO2 to be maintained and receive less interference from the anatomical DS of the upper respiratory tract, however, supporting evidence is still lacking.^17^ Thus, the use of a single‐sided TC appears to be superior to reducing the upper respiratory DS and provides smooth oxygen delivery than other devices/measures for patients who are obese or have sleep apnea.

There may be some risks for dislocation of the TC in cases where EUS scope was in practice especially. The scope moves sometimes in a forward‐and‐backward manner while observational EUS was performed, which could cause friction between the endoscope and the catheter. Consequently, the dislocation of the tube could happen. Although there was no incidence of the dislocation of the catheter in this study, endoscopists should pay attention to this.

There were further several limitations to this study: (i) it was a single‐center study and the findings need to be validated in a multi‐institutional study, (ii) the endoscopy procedures performed in the study on patients were limited to PBE, (iii) the data were short‐term results, (iv) the participants were relatively healthy individuals and the study population did not include patients with sinusitis, and so forth, in whom it is difficult to introduce a TC, and (v) PO2 data (partial pressure of oxygen evaluated in the artery) was not studied. SpO2 was used as an alternative, which might be lacking in accuracy. Additionally, as the respiratory crisis may be often occurred by central respiratory suppression by anesthetic drugs and dropping tongue roots, the benefit of this single‐sided TC could be limited to conserving the airway.

In conclusion, our feasibility study indicated that oxygen delivery through this single‐sided TC, a non‐invasive and patient‐friendly device, allowed safe and efficient oxygen delivery in patients undergoing even therapeutic PBE by decreasing the anatomical DS in the nasal, oral, and pharyngeal cavities.

## CONFLICT OF INTEREST

The authors declare no conflict of interest.

## ETHICS STATEMENT

Ethics approval was granted by the NTT medical center Tokyo Insititutional Review Board (ID:77567).

## FUNDING INFORMATION

None.
